# Developing a Cobalt Phosphide Catalyst with Combined Cobalt Defects and Phosphorus Vacancies to Boost Oxygen Evolution Reaction

**DOI:** 10.3390/ma17184647

**Published:** 2024-09-22

**Authors:** Weihua Ou, Ligui Li, Wei Zhou, Minzhe Chen, Chuheng Zhu, Xiaoyan Zhu, Ke Yuan

**Affiliations:** New Energy Research Institute, College of Environment and Energy, South China University of Technology, Guangzhou 510006, China

**Keywords:** defect engineering, cobalt defects, phosphorus vacancies, oxygen evolution reaction

## Abstract

Defect engineering, by adjusting the surface charge and active sites of CoP catalysts, significantly enhances the efficiency of the oxygen evolution reaction (OER). We have developed a new Co_1−x_P_v_ catalyst that has both cobalt defects and phosphorus vacancies, demonstrating excellent OER performance. Under both basic and acidic media, the catalyst incurs a modest overvoltage, with 238 mV and 249 mV needed, respectively, to attain a current density of 10 mA cm^−2^. In the practical test of alkaline electrocatalytic water splitting (EWS), the Co_1−x_P_v_ || Pt/C EWS shows a low cell voltage of 1.51 V and superior performance compared to the noble metal-based EWS (RuO_2_ || Pt/C, 1.66 V). This catalyst’s exceptional catalytic efficiency and longevity are mainly attributed to its tunable electronic structure. The presence of cobalt defects facilitates the transformation of Co^2+^ to Co^3+^, while phosphorus vacancies enhance the interaction with oxygen species (*OH, *O, *OOH), working in concert to improve the OER efficiency. This strategy offers a new approach to designing transition metal phosphide catalysts with coexisting metal defects and phosphorus vacancies, which is crucial for improving energy conversion efficiency and catalyst performance.

## 1. Introduction

With economies and societies advancing and the reserves of conventional fossil fuels depleting, the need for sustainable energy conversion and storage technologies is growing steadily [1]. Hydrogen, generated through water electrolysis, acts as a pristine and concentrated energy source, holding promise to supplant fossil fuels in the future, thereby realizing the vision of sustainable energy development and minimizing greenhouse gas emissions [2,3,4]. Regrettably, the anodic OER is characterized by high overpotentials due to the sluggish nature of the complex four-electron transfer process. which seriously hampers the integral operational effectiveness and limits the large-scale commercialization of water electrolyzers [5]. High-priced electrocatalysts that are based on precious metals like RuO_2_ and IrO_2_ continue to be the most efficient in both acidic and alkaline environments [6]. However, the scalability of their production is hindered by the high cost of materials, the scarcity of these resources, and doubts about their durability during operational periods. Significant advancements have been made in crafting non-precious metal catalysts with robust catalytic efficacy in the past few years, encompassing various transition metal-derived materials such as nitrides, sulfides, oxides, and phosphides [7,8,9,10]. Cerium-based catalysts show great potential in OER due to their unique electronic structure and REDOX properties [11]. For example, Cerium-based metal–organic framework (MOF)-derived electrocatalysts can significantly improve their conductivity and stability and thus exhibit excellent electrocatalytic performance in OER [12]. Cobalt spinel structures, known for their adjustable cation oxidation states, abundant reactive oxygen, and economic benefits [13,14,15], include cobalt phosphide (CoP), which is emerging as a substitute for expensive platinum-group metal catalysts due to its significant REDOX properties, superior electrical conductivity, and pronounced OER performance [16,17]. Nevertheless, the efficiency of CoP catalysts is often impeded by poor electrical conductivity, suboptimal active site engagement, and restricted electron transfer, limiting its potential for commercial applications [18,19]. Therefore, finding solutions to enhance the electrocatalytic activity of CoP is essential in improving the overall performance of the OER.

To enhance the catalytic performance of CoP, researchers commonly employ interfacial regulation techniques such as doping [20], support engineering [21], and the introduction of vacancies [22]. These strategies are intended to alter the catalyst’s molecular configuration and electronic traits, which in turn boost its effectiveness in electrocatalysis. Among these methods, defect engineering, due to its low coordination number, can cause a redistribution of surface charge and may produce a large amount of delocalized electrons, inducing the catalyst to generate new catalytic sites [23,24]. Hence, introducing defects is viewed as a potent tactic for manipulating the surface charge distribution and maximizing the reactivity at catalytic sites. Past research has shown that metal defects can lead to substantial electronic redistribution, providing extra active sites for catalysis [25,26]. Concurrently, anion vacancy defects in metal compounds can effectively alter the electronic configurations of neighboring metal atoms, which in turn affects the energy thresholds for reactants and intermediates [27]. Additionally, phosphorus vacancy (P_v_) can reduce the hybridization of P 2p orbitals and increase the electron density around adjacent Ni and P atoms [28]. To the best of our awareness, there is a dearth of research on catalysts with concurrent vacancies in both metal and phosphorus, and the special catalytic characteristics and mechanisms of these catalysts with coexisting defects remain largely uncharted territory.

In this work, glycerol cobalt (CoGly) precursors were synthesized via hydrothermal synthesis and cobalt-deficient cobalt oxide was obtained through calcination at various temperatures. Subsequently, through the implementation of low-temperature phosphidation and H_2_ reduction treatment, we successfully synthesized a catalytic material denoted as Co_1−x_P_v_, possessing both cobalt defects and phosphorus vacancies. This Co_1−x_P_v_ catalyst exhibits superior performance in the OER compared to Co_1−x_P (cobalt defects only), CoP_v_ (phosphorus vacancies only), and pristine CoP under both alkaline and acidic conditions.

## 2. Experimental Section

### 2.1. Catalyst Preparation

**Figure 1:**
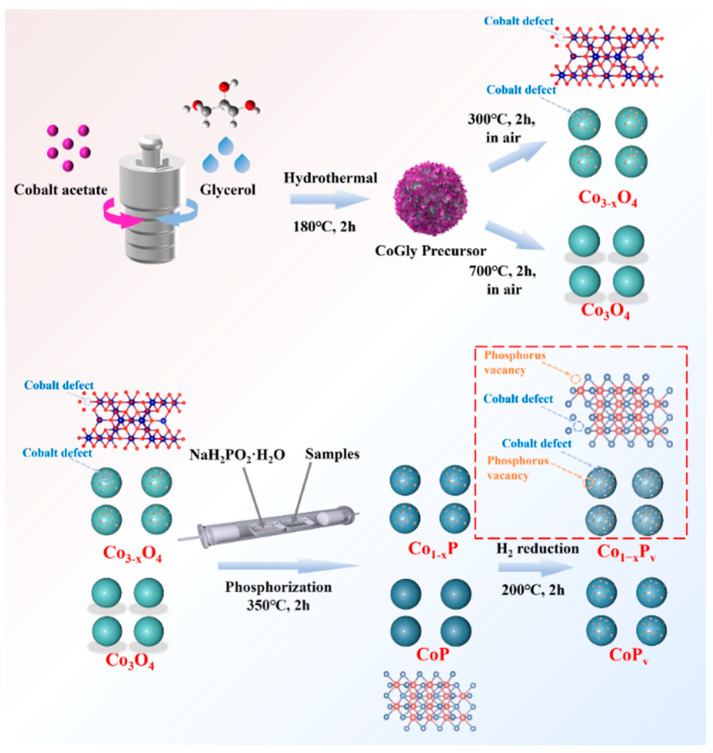
The synthesis methods for Co_1−x_P_v_, CoP_v_, Co_1−x_P, and CoP catalysts.

The catalysts were fabricated using a solvothermal synthesis coupled with a high-temperature phosphation process, followed by hydrogen reduction treatment, as illustrated in Figure 1. A mixture was prepared by dissolving 1.0 g of cobalt acetate tetrahydrate into 30 milliliters of glycerol, followed by 60 min of mixing using a magnetic stirrer within a 100 mL Teflon-coated autoclave. The temperature was then raised to 180 °C and sustained for a duration of 2 h. After centrifugation, the precipitates were isolated and cleaned with ethanol in triplicate. They were later dried at 60 °C for a full night. The powder was then annealed in air for 2 h at both 300 °C and 700 °C, applying a gradual heating increment of 5 °C min^−1^. The resulting products were named Co_3−x_O_4_ and Co_3_O_4_, respectively. The samples of Co_3−x_O_4_ and Co_3_O_4_ were subjected to a low-temperature phosphorization process at 350 °C for a duration of 2 h in an inert atmosphere, using sodium phosphomonohydrate, which resulted in the synthesis of Co_1−x_P and CoP. Continuing the process, the samples were further treated in a 10% H_2_ in Ar gas mixture (at a flow rate of 250 sccm) at 200 °C for 60 min, with a gradual temperature ramp of 2 °C min^−1^, culminating in the production of Co_1−x_P_v_ and CoP_v_.

### 2.2. Structure Characterization

The analysis of X-ray diffraction (XRD) was performed using a Bruker D8-Advance diffractometer (Billerica, MA, USA) equipped with Cu Ka radiation. To examine the surface morphology and microstructure of the prepared samples, we utilized a field emission scanning electron microscope (FE-SEM, S-4800, Hitachi, Tokyo, Japan) and transmission electron microscopy (TEM, Talos F200x with an acceleration voltage of 200 kV, Thermo Fisher Scientific, Waltham, MA, USA). The chemical compositions were investigated through X-ray photoelectron spectroscopy (XPS) employing Al Ka X-rays on the Phi X tool instrument (Ulvac, Chigasaki, Japan). For accurate measurements, we adjusted the binding energy of C1s to 284.6 eV. Raman spectrum analysis covered a wave number range of 100 to 1000 cm^−1^ and was conducted using a LabRAM HR Evolution instrument (HORIBA, Kyoto, Japan). To determine the specific surface area and pore size distribution, nitrogen adsorption–desorption at 77 K was performed using an ASAP 2010 instrument (Micromeritics, Norcross, GA, USA). We employed the Brunell–Emmett–Teller (BET) model equation for calculating a specific surface area while analyzing pore size distribution utilizing BJH and DFT models. The characterization of the unpaired electron state in the prepared samples was carried out using Electron Paramagnetic Resonance (EPR, Bruker EMXplus).

### 2.3. Electrode Preparation

A 5 mg portion of the catalyst was mixed with a solution comprising 80 μL of water, 900 μL of ethanol, and 20 μL of 0.05 weight percent Nafion solution to create a homogeneous suspension. This was sonicated for the duration of 1 h to create a uniform ink. For the electrode preparation, 10 μL of this ink was spread over a pre-polished glassy carbon electrode measuring 3 mm in diameter, which corresponded to a loading of 0.28 mg cm^−2^. The final step involved drying the electrode under a heat lamp for 5 min to ensure the solvent had fully evaporated.

### 2.4. Electrochemical Measurement

The CHI-660E potentiostat (CH Instruments, Bee Cave, TX, USA) was employed for conducting all OER experiments. KOH at a concentration of 1.0 M acted as the electrolyte for the alkaline media, whereas H_2_SO_4_ at 0.5 M concentration was applied for the acidic environment. We assembled a three-electrode system using a glassy carbon electrode loaded with a catalyst as the working electrode and a graphite rod as the counter electrode. For alkaline conditions, the reference electrode was a Hg/HgO electrode, and for acidic conditions, it was a Hg/Hg_2_SO_4_ electrode. Linear sweep voltammetry (LSV) tests were performed at a scanning rate of 5 mV s^−1^. The EIS measurements were conducted over a frequency range from 100 kHz down to 0.01 Hz, using a signal amplitude of 5 mV. Cyclic voltammetry was performed in the non-Faradaic region to determine the double-layer capacitance (C_dl_).

## 3. Results and Discussion

### 3.1. Morphology Analysis

**Figure 2:**
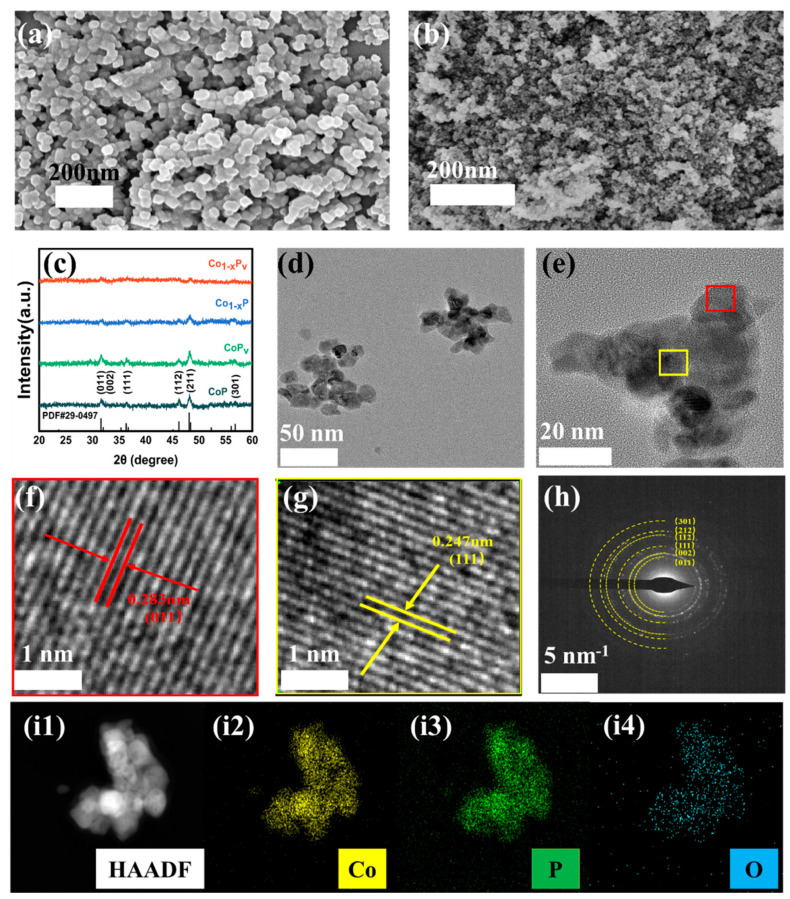
SEM images of (**a**) CoP (**b**) Co_1−x_P_v_. (**c**) XRD patterns of Co_1−x_P_v_, CoP_v_, Co_1−x_P, and CoP catalysts. (**d**) TEM image and (**e**–**g**) HRTEM images. (**h**) The SAED image and (**i1**–**i4**) EDX mappings of Co_1−x_P_v_.

The XRD pattern (Figure S1a) indicates the synthesis of CoGly (II) with low crystallinity. The SEM image (Figure S1b) demonstrates an abundance of numerous tiny nanosheets within the CoGly structure, which are likely the outcome of a tiered crystalline structure, characterized by (···Co-O-Co-O···) linkages and -O-C-C(C-OH)-O- terminal groups [29]. The multi-layer frame promotes the appearance of cobalt defects, and their concentration can be directly controlled by adjusting the heat treatment temperature [30,31]. Figure 1 shows that a considerable amount of Co-deficient Co_3−x_O_4_ can be synthesized by thermally treating the CoGly (II) precursor at 300 °C in air. Subsequently, the phosphorization of the sample at 350 °C for 2 h under inert conditions with sodium phosphomonohydrate induced cobalt defects in the Co_1−x_P, enhancing its catalytic properties. Following the phosphorization, a hydrogen reduction at 200 °C was applied to create a rich surface of phosphorus vacancies alongside cobalt defects within the Co_1−x_P_v_, thus enhancing the sample’s catalytic activity. In contrast, the crystalline Co_3_O_4_ was created by calcining the precursor at 700 °C in air, then subjected to phosphorization at 350 °C for 2 h in an inert atmosphere using sodium phosphomonohydrate to produce pristine CoP. Phosphorus vacancy CoP_v_ was produced by following H_2_ reduction (200 °C). The SEM images reveal that Co_1−x_P_v_ displays a nano-particle morphology akin to that of the original CoP, as illustrated in Figure 2a,b. However, Co_1−x_P_v_ was annealed at relatively low temperatures (300 °C compared to 700 °C), and the particle size diminished. The reason for this lies in the strong correlation between the particle size of the material and the sintering temperature [32]. As the sintering temperature is reduced during the synthesis process, the particle size of the material diminishes accordingly. Additionally, the incorporation of phosphorus vacancies contributes to an enhanced surface roughness of the material [28]. The XRD patterns (Figure 2c) substantiate the synthesis of cobalt phosphide, evidenced by the distinctive peaks located at angles of 31.6, 32.0, 36.3, 46.2, 48.1, and 56.8, which can be indexed to (011), (002), (111), (112), (211), and (301) reflections, respectively, corresponding to the Fd3m CoP structure (JCPDS no. 29-0497). Compared to the original sample, Co_1−x_P_v_ exhibits weaker and less sharp diffraction peaks, indicating a decrease in crystallinity and smaller grain size. The detection of a minor shift in the diffraction peaks towards lower angles as the calcination temperature rises suggests a reduction in the interplanar spacing at lower temperatures, consistent with Bragg’s law. The likely reason for this lattice shrinkage is attributed to the creation of defects in the low-temperature calcination phase [33].

The TEM image depicted in Figure 2d illustrates that Co_1−x_P_v_ possesses a nano-particle morphology, with an approximate diameter of 50 nm. This nano-particle structure facilitates selective control over the catalytic reaction process by allowing for the manipulation of its surface properties and crystal structure The HR-TEM image is depicted in Figure 2e, with the boxed annotation directing to the regions in Figure 2f,g. This annotation reveals that the interplanar spacings in Co_1−x_P_v_ are 0.247 nm and 0.283 nm, corresponding to the (111) and (011) crystallographic planes of CoP, respectivelyMoreover, the SAED pattern (Figure 2h) exhibits clear diffraction rings, which confirm the polycrystalline structure of the prepared Co_1−x_P_v_. Additionally, the EDX elemental distribution depicted in Figure 2i1–i4 demonstrates the uniform distribution of Co, O, and P across the entire composite material.

**Figure 3:**
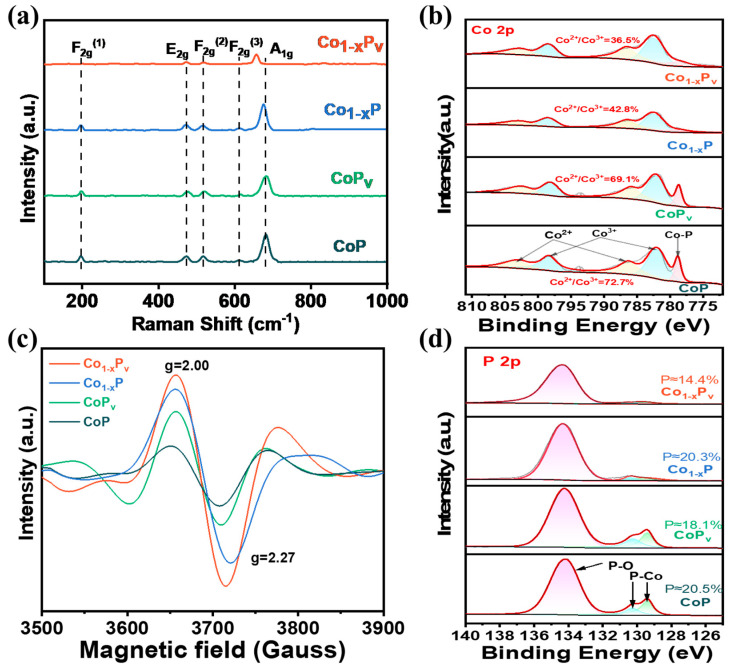
(**a**) Raman spectrums, (**b**) Co 2p XPS spectrum, (**c**) EPR patterns, and (**d**) P 2p XPS spectrum of Co_1−x_P_v_, CoP_v_, Co_1−x_P, and CoP Catalysts.

Utilizing Raman spectroscopy for further structural analysis, the resultant samples from the described synthesis steps display five specific Raman peaks indicative of CoP, as observed in Figure 3a. Raman analysis identified five distinct peaks located at 196, 472, 516, 610, and 680 cm^−1^, each corresponding to the specific symmetries of F_2g_^(1)^, E_2g_, F_2g_^(2)^, F_2g_^(3)^, and A_1g_ [34,35]. In nanomaterials, due to the effects of size and surface effects, defects such as dislocations, vacancies, or grain boundaries in the samples can alter the vibration modes of phonons, thereby affecting the vibrational frequencies related to lattice vibrations or chemical bonds. This may lead to the emergence of new Raman vibrational peaks or changes in the position and intensity of existing peaks [36]. The A_1g_ symmetry in Co_1−x_P_v_ demonstrates a notable downward shift, indicative of a lower degree of short-range order. Furthermore, the F_2g_^(1)^ peak at 196 cm^−1^ has a noticeable reduction in intensity, pointing to a lessened long-range order [37]. These reductions in range order suggest an abundance of defect sites within the material [38], especially in Co_1−x_P_v_.

Employing N_2_ adsorption–desorption measurements, the specific surface area and porosity of the synthesized samples are assessed (as shown in Figure S2a–d). The type-IV isotherm patterns, complete with distinct hysteresis loops within the 0.5 to 1.0 relative pressure span, denote the interplay of mesoporous and microporous architectures within the materials [39]. The presence of both mesoporous and microporous frameworks can improve the rate of mass exchange between the material and its surroundings [40]. This means that during catalytic reactions and other processes, the material can more effectively facilitate the transfer of electrons/ions, thereby enhancing reaction efficiency. Additionally, the coexistence of mesoporous and microporous structures can improve the stability and mechanical strength of the material [41], which enables the material to better withstand external environmental influences, prolong its lifespan, and maintain good performance. The specific surface area of Co_1−x_P_v_ was measured using the BET technique and is 89.34 m^2^ g^−1^, and the mean pore volume is 3.17 cm^3^ g^−1^. The hydrogen reduction process is noted to cause an increase in the material’s surface area as well as the dimensions of its pores, likely owing to a decrease in the catalyst’s crystalline order and the resultant increase in surface irregularity [28], and such an increase is favorable for the revelation of active sites necessary for the electrocatalytic reaction [42].

Further investigation of the samples’ electronic configurations of Co and P was performed using XPS, as shown in Figure 3b,d. Figure 3b shows a main peak located at 778.7 eV, which corresponds to Co–P bonds. The spectral features at 786.4 eV and 802.6 eV are indicative of the presence of Co^2+^, and the peaks found at 782.3 eV and 798.3 eV are attributed to Co^3+^ [43,44]. The binding energy for Co^3+^ is found to be lower than that for Co^2+^, a result of its unique electronic structure, with Co^2+^ (3d^5^4s^2^) being more stable than Co^3+^ (3d^5^4s^1^) [45,46]. The detection of Co^3+^ is presumed to originate from the oxidation process of Co^2+^ in the nascent stage of nanostructure creation. Based on the semi-quantitative evaluation from XPS, among them, the relative atomic ratio of Co^2+^/Co^3+^ is 36.5% for Co_1−x_P_v_, lower than that of Co_1−x_P (42.8%), CoP_v_ (69.1%), and CoP (72.7%). The data reveal a significant abundance of Co^3+^ species within Co_1−x_P_v_, accompanied by an increased level of phosphorus vacancy concentration. It is worth noting that comparing various samples reveals that the presence of cobalt defects can significantly reduce the content of Co–P bonds and the relative atomic ratio of Co^2+^/Co^3+^. The P 2p XPS spectrum in Figure 3d shows peaks at 129.5 eV and 130.4 eV corresponding to the low oxidation state phosphorus in CoP for P 2p_3/2_ and P 2p_1/2_, respectively. The higher binding energy peak for P–O is associated with oxidized species such as PO_4_^3−^ or P_2_O_5_^−^ [47]. The content of the Co–P bonds in Co_1−x_P_v_ are significantly lower than the other samples, which corresponds to the Co spectrum. Furthermore, from the wide-scan XPS spectrum (Figure S3), the P atom percentage in Co_1−x_P_v_ is noted to be 14.4%, which is lower compared to Co_1−x_P (20.3%), CoP_v_ (18.1%), and CoP (20.5%), a result of the hydrogen reduction treatment, indicating the creation of phosphorus vacancies (P_v_). The decrease in the P element content leads to a further increase in the electron density around it, consequently enhancing coupling with the Co element [48]. Electron paramagnetic resonance (EPR) is another direct method to survey phosphorus vacancies and cobalt defects of the samples. As depicted in Figure 3d, EPR at low temperatures was utilized to discern the presence of unpaired electrons and vacancies on the catalyst’s exterior and beneath the surface layers. Each sample displayed two distinct signals at g values of 2.27 and 2.00, likely due to the phosphorus vacancies on the surface [49] and the presence of unpaired electrons in the Co d orbitals within the near-surface layer [50]. The EPR signal intensity of four catalysts follows the order of Co_1−x_P_v_ > CoP_v_ > Co_1−x_P > CoP. The more saturated state correspondingly possessed fewer unpaired electrons [51]. This trend demonstrates that the introduction of Co defects and phosphorus vacancies, especially coexisting vacancies, is beneficial for changing the spin state of Co and P atoms, which leads to an increase in unliganded electrons. The result is consistent with the XPS spectra and Raman findings, substantiating the role of cobalt defects and phosphorus vacancies in altering the electronic properties of the catalyst.

### 3.2. Electrochemical Oxygen Evolution Reaction Performance

We began by using LSV to evaluate the OER catalytic capabilities of Co_1−x_P_v_, Co_1−x_P, CoP_v_, and CoP, recording their polarization curves in a 1.0 M KOH electrolyte.

**Figure 4:**
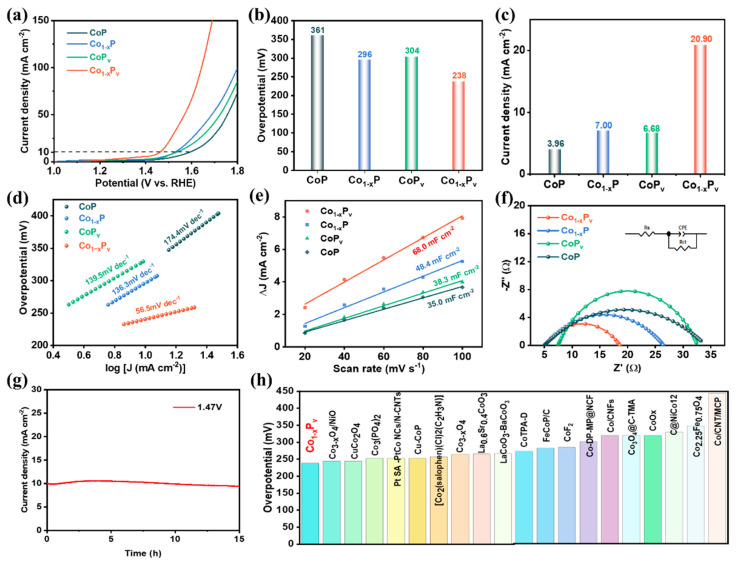
(**a**) The LSV, (**b**) the comparison of overpotential at 10 mA cm^−2^, (**c**) current density at 270 mV (**d**), Tafel plots, (**e**) current density differences plotted against scan rates, (**f**) Nyquist plots (**g**), i-t curve of catalysts in 1.0 M KOH, and (**h**) the comparison of overpotentials at η = 10 for Co_1−x_P_v_ and reported cobalt-based catalysts.

The LSV curves, accompanied by histograms that illustrate the overpotential, are shown in Figure 4a,b, which indicated that the overpotential of Co_1−x_P_v_ at 10 mA cm^−2^ is 238 mV, significantly lower than those of Co_1−x_P (296 mV), CoP_v_ (304 mV), and CoP (361 mV). Moreover, as shown in Figure 4c, the current densities at an overpotential of 270 mV for the four catalysts are presented. Co_1−x_P_v_ stands out with the highest current density of 20.9 mA cm^−2^, which is nearly six times the value of the unmodified CoP at 3.96 mA cm^−2^ and about three times the current density of both Co_1−x_P and CoP_v_, which are 7.00 and 6.68 mA cm^−2^, respectively. Further evaluation of activity is evident from the Tafel plots (Figure 4d). The calculated Tafel slope of Co_1−x_P_v_, characterized by an abundance of cobalt defects and phosphorus vacancies, is roughly 56.5 mV dec^−1^. This value significantly contrasts with Co_1−x_P (139.5 mV dec^−1^), CoP_v_ (136.3 mV dec^−1^), and CoP (174.4 mV dec^−1^). The proposition that incorporating cobalt defects and phosphorus vacancies substantially boosts the catalytic activity of CoP in OER is a notable one. In addition, the evaluation of the performance of electrocatalysts relies heavily on the measurement of the electrochemical active area (ECSA), which represents the effective surface of the electrochemical reaction and is an important parameter to measure the performance of electrocatalysts. It is worth noting that there is a positive correlation relationship between double-layer capacitors (C_dl_) and ECSA. The measurement of C_dl_ helps to understand the extent to which the active site influences the efficiency of the electrocatalyst and can be used as a quantitative method for ECSA, as shown in Figure 4e and Figure S4. This investigation aims to explore the impact of introducing cobalt defects and phosphorus vacancies on the OER performance of CoP. This can expose the extent of the active sites that are pivotal to the electrocatalyst’s effectiveness. Impressively, Co_1−x_P_v_ exhibited a higher value of 68.0 mF cm^−2^ compared to Co_1−x_P (48.4 mF cm^−2^), CoP_v_ (38.3 mF cm^−2^), and CoP (35.0 mF cm^−2^), which suggests the largest exposure of catalytic active sites and abundant active surface sites, contributing to the excellent electrocatalytic OER performance in LSV. The proliferation of active sites is likely due to the increased surface roughness caused by phosphorus vacancies and/or the shift from a single-crystalline to a polycrystalline state, leading to diminished crystallinity. EIS is utilized to uncover the inherent characteristics of interfacial charge transfer and the dynamics of charge movement within electrocatalysts during the OER. In Figure 4f, the presence of a semicircle at low frequencies indicates that the electrode reaction is primarily governed by kinetic barriers, not by limitations in mass transfer [52]. As shown in the Nyquist plot, the Co_1−x_P_v_ material demonstrates a smaller semicircle diameter compared to other materials, indicating superior charge transfer performance. In this component, the values of Rct are 13.6, 21.4, 25.0, and 28.2 Ω for Co_1−x_P_v_, Co_1−x_P, CoP_v_, and CoP, respectively. The findings indicate that Co_1−x_P_v_ possesses lower charge transfer resistance, with Co vacancies and P deficiencies enhancing electron transfer and augmenting the electron density at active sites, which reduces the interfacial impedance and facilitates quicker electron transfer, thereby further enhancing the catalytic activity for the OER. These results are in line with the findings from previous scholarly articles. Assessing stability over an extended period at a constant current is vital for evaluating the electrocatalytic performance with practical applications in mind. The Co_1−x_P_v_ sample, which exhibited the highest OER performance, underwent a durability assessment through chronoamperometry. The test involved monitoring the current density over time at a fixed voltage of 1.47 V, corresponding to a current density of 10 mA cm^−2^, for a duration of 15 h, as depicted in Figure 4g. The absence of a notable drop in current density throughout sustained electrolysis suggests excellent catalytic stability for the OER. The high activity and great stability of Co_1−x_P_v_ make it a promising candidate for broad applications. Crucially, Co_1−x_P_v’_s electrocatalytic performance for the OER is on par with, and in some cases surpasses, many of the latest Co-based catalysts reported for use in alkaline environments [53,54] (Figure 4h). Thus, the discussed results firmly establish Co_1−x_P_v_ as an outstanding catalyst for the OER in alkaline conditions.

**Figure 5:**
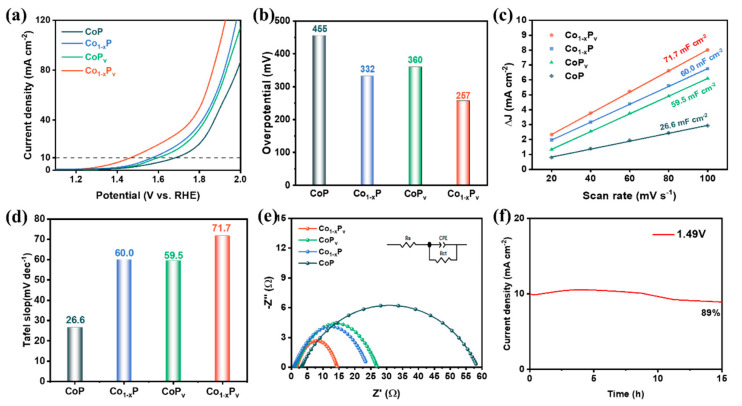
(**a**) The LSV, (**b**) the corresponding histograms of overpotentials at 10 mA cm^−2^, (**c**) current density differences plotted against scan rates, (**d**) histograms of electrochemical double layer capacitance, (**e**) Nyquist plots, and (**f**) *i*-*t* curve of catalysts in 0.5 M H_2_SO_4_.

In comparison with the abundance of OH^−^ in alkaline media, in acidic media, there are additional steps and kinetic barriers due to the adsorption and dissociation of water at active sites [55]. Moreover, under highly corrosive and oxidative acidic conditions, catalysts are prone to corrosion and dissolution, so the catalytic activity of general catalyst materials in acidic media is relatively poor. Despite this, the Co_1−x_P_v_ catalyst prepared in this study also exhibits good catalytic activity. We assessed the OER activity of the materials in a 0.5 M H_2_SO_4_ solution. The LSV curves from the 0.5 M H_2_SO_4_ electrolyte tests reveal that Co_1−x_P_v_ outperforms Co_1−x_P, CoP_v_, and CoP in terms of catalytic activity across the examined voltage spectrum. Co_1−x_P_v_ attains a current density of 10 mA cm^−2^ at a notably reduced overpotential of 257 mV, outperforming Co_1−x_P (332 mV), CoP_v_ (360 mV), and CoP (455 mV). In addition, we also calculated the C_dl_ of each catalyst, as shown in Figure 5c,d and Figure S5. In an acidic medium, the C_dl_ of Co_1−x_P_v_ was 71.7 mF cm^−2^, which is greater than that of Co_1−x_P, CoP_v_, and CoP. Co_1−x_P_v_ exhibits a larger electrochemically active area, thereby providing more active sites for the OER. By applying EIS technology, this study analyzed the characteristics of interface charge transfer and the charge transport dynamics of the electrocatalyst. In Figure 5e, based on the simulation of the Nyquist plot, the Rct (charge transfer resistance) values for Co_1−x_P_v_, Co_1−x_P, CoP_v_, and CoP are 12.70, 35.56, 22.54, and 54.92 Ω, respectively. This indicates that Co_1−x_P_v_ has a smaller charge transfer resistance under acidic conditions, and the charge transport at its interface is more conducive to the electrocatalytic OER. The electrochemical durability of Co_1−x_P_v_ was also tested using chronoamperometry, and the time-dependent current density at a constant voltage of 1.49 V required to achieve a current density of 10 mA cm^−2^ is shown in Figure 5f. During continuous electrolysis, although the current density decreased, it still maintained 89% of the original current density, indicating that Co_1−x_P_v_ also has good catalytic stability in the acidic OER process. In acidic environments, the catalyst’s stability is crucial for maintaining long-term activity. Co_1−x_P_v_ exhibits a coexistence of mesoporous and microporous structures, which confers excellent chemical stability and resistance to corrosion and dissolution under acidic conditions, thereby preserving its catalytic activity.

**Figure 6:**
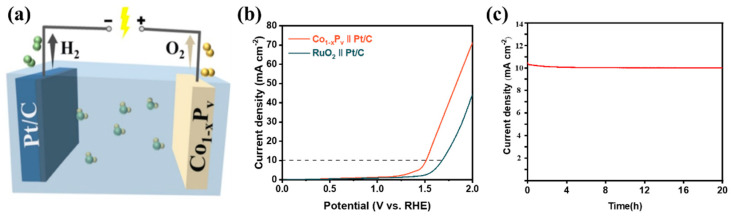
(**a**) Diagram of the water electrolysis process, (**b**) polarization curves of Co_1−x_P_v_ || Pt/C and RuO_2_ || Pt/C in 1.0 M KOH, (**c**) the *i*-*t* curve of Co_1−x_P_v_ || Pt/C EWS.

An effective OER catalyst should facilitate efficient water electrolysis, enabling superior electrocatalytic water splitting (EWS) [56]. In this context, the water-splitting electrolyzer in a 1.0 M KOH solution is constructed with Pt/C serving as the cathode and Co_1−x_P_v_ as the anode, as illustrated in the diagram of Figure 6a. As a comparison, utilize Pt/C as the cathode and RuO_2_ as the anode to measure the performance of electrolyzed water. The LSV curves for EWS depicted in Figure 6b show that Co_1−x_P_v_ requires only 1.51 V to achieve a current density of 10 mA cm^−2^, demonstrating superior performance compared to the Pt/C || RuO_2_-based system, which needs 1.67 V to reach the same current density. Furthermore, the *i*-*t* test for EWS, as shown in Figure 6c, indicates that the Pt/C || Co_1−x_P_v_ system exhibits excellent durability, with minimal current density loss after 20 h operation, underscoring the potential of Co_1−x_P_v_ for hydrogen generation through practical EWS applications.

Following an extended stability evaluation, the Co_1−x_P_v_ sample was subjected to XPS analysis to determine alterations in the surface chemical composition and the oxidation state. The detailed Co 2p core level XPS spectrum post-OER testing is presented in Figure S5a. The Co–P bond signal, which was present in the initial OER XPS analysis, vanished after the exposure. The ratio of Co^2+^ to Co^3+^ atoms is 27.2%, suggesting that the enhanced presence of Co^3+^ ions is likely responsible for the improved OER activity. Figure S5b displays the P 2p XPS analysis results for the Co_1−x_P_v_ sample. The intensity of the P–O bond peak has notably risen, signifying the emergence of phosphate groups due to surface oxidation. Concurrently, the reduction in the Co–P bond peak intensity corroborates the shift towards the formation of oxyhydroxides. Figure S5c illustrates the deconvoluted O 1s spectrum of Co_1−x_P_v_, which resolves into three distinct peaks. The peak at 529.4 eV signifies the presence of oxygen atoms in metal bonds, indicating the formation of metal oxyhydroxides during the OER. The prominent peak observed at 530.9 eV is indicative of the OH^−^ groups, which are associated with the formation of metal hydroxides. Additionally, the peak at 532.7 eV can be attributed to the presence of water molecules that are physically adsorbed onto the surface.

The oxygen evolution reaction (OER) involves four fundamental stages, each requiring the creation of intermediates, such as *OH, *O, and *OOH, along with the release of oxygen molecules from the catalyst’s active sites [57,58,59]. Studies on cobalt phosphide, cobalt chalcogenides (such as CoS_2_, CoTe_2_, and CoSe_2_), and cobalt oxide catalysts have consistently demonstrated that these materials transform into cobalt oxyhydroxides under alkaline environments during the OER [60,61,62]. Previous studies have indicated that Co^3+^ may act as the catalytically active center of OER [17,63]. In this study, CoP underwent a transformation to CoOOH under highly alkaline conditions during the OER, as confirmed by XPS analysis. Co continues to function as the key catalytic site, with the electrons near the P_v_ boosting conductivity, which in turn aids in the creation of *OH, *O, and *OOH intermediates at the cobalt locations [64]. The two factors mentioned are central to achieving high-efficiency OER transport kinetics. Based on the previous discussion, the underlying mechanism of Co_1−x_P_v_ may be driven by the suitable coexistence of Co defects and P vacancies. The Co defects facilitate the transformation of Co^2+^ to Co^3+^, and the P vacancies lead to the formation of undercoordinated cobalt sites, which in turn enhance the ideal binding energy for intermediates. This dual effect synergistically enhances the OER performance and diminishes the overpotential.

## 4. Conclusions

In conclusion, this research presents a straightforward method for synthesizing cobalt phosphide (Co_1−x_P_v_) catalysts with thermal and hydrogen reduction treatment of glycerol cobalt precursors, resulting in catalysts with cobalt and phosphorus vacancies. These vacancies significantly enhance the catalyst’s OER activity under a wide range of pH conditions, increasing active sites and accelerating electron transfer through electronic modulation and morphological adjustment. XPS analysis shows that cobalt vacancies promote the transformation from Co^2+^ to Co^3+^, while the disordered electrons near phosphorus vacancies enhance electrical conductivity and facilitate the formation of more oxygen intermediates (*OH, *O, *OOH) at cobalt sites, effectively promoting the binding of these intermediates with uncoordinated Co. These improvements notably reduce the overpotential for OER, offering a new strategy for designing efficient transition metal phosphide catalysts.

## Data Availability

The original contributions presented in the study are included in the article/supplementary material, further inquiries can be directed to the corresponding author.

## Supplementary Materials

The following supporting information can be downloaded at: https://www.mdpi.com/article/10.3390/ma17184647/s1, Figure S1: (a) XRD pattern and (b) SEM image of CoGly; Figure S2: The Nitrogen adsorption–desorption isotherms of (a) CoP, (b) CoP_v_, (c) Co_1−x_P, and (d) Co_1−x_P_v_, where the inserts are the relevant pore size distribution; Figure S3: The wide-scan XPS spectrum of (a–b) CoP, CoP_v_, Co_1−x_P, Co_1−x_P_v_; Figure S4: CV curves of (a–d) CoP, Co_1−x_P, CoP_v_, Co_1−x_P_v_ in the non-Faraday region of 1.075 V–1.175 V vs. RHE with various scan rates (20, 40, 60, 80, and 100 mV s^−1^) for OER in 1.0 M KOH; Figure S5: XPS spectrums of (a) Co 2p (b) P 2p, and (c) O 1s of Co_1−x_P_v_ after *i*-*t* test; Figure S6: CV curves of (a–d) CoP, Co_1−x_P, CoPv, and Co_1−x_Pv in the non-Faraday region of 1.05 V–1.15 V vs. RHE with various scan rates (20, 40, 60, 80, and 100 mV s^−1^) for OER in 0.5 M H_2_SO_4_; Table S1: EDX result of Co_1−x_P_v_. Reference [65] is cited in the supplementary materials.
