# Supplementary material for: Developing a Cobalt Phosphide Catalyst with Combined Cobalt Defects and Phosphorus Vacancies to Boost Oxygen Evolution Reaction
Source: Materials (Basel). 2024 Sep 22;17(18):4647. doi: 10.3390/ma17184647 (PMC11433320; doi:10.3390/ma17184647)
Supplement: Supplementary file 1 [file materials-17-04647-s001.zip › materials-3182621-supplementary.pdf]

Supplementary Material

Developing a Cobalt Phosphide Catalyst with Combined Cobalt Defects and Phosphorus Vacancies to Boost Oxygen Evolution Reaction

Weihua Ou, Ligui Li *, Wei Zhou, Minzhe Chen, Chuheng Zhu, Xiaoyan Zhu and Ke Yuan

New Energy Research Institute, College of Environment and Energy, South China University of Technology, Guangzhou 510006, China.

* Corresponding author. E-mail: esguili@scut.edu.cn

Contents

Figure S1. (a) XRD pattern and (b) SEM image of CoGly.

Table S1. EDX result of $Co_{1-x}P_v$.

Figure S2. The Nitrogen adsorption-desorption isotherms of (a) CoP, (b) CoP_v, (c) $Co_{1-x}P$ and (d) $Co_{1-x}P_v$, where the inserts are the relevant pore size distribution.

Figure S3. The wide-scan XPS spectrum of (a-b) CoP, CoP_v, $Co_{1-x}P$, $Co_{1-x}P_v$.

Figure S4 CV curves of (a-d) CoP , $Co_{1-x}P$, CoP_v , $Co_{1-x}P_v$ in the non-Faraday region of 1.075 V-1.175 V vs. RHE with various scan rates (20, 40, 60, 80 and 100 mV s^{-1}) for OER in 1.0 M KOH.

Figure S5 XPS spectrums of (a) Co 2p (b) P 2p and (c) O 1s of $Co_{1-x}P_v$ after i-t test.

Figure S6 CV curves of (a-d) CoP , $Co_{1-x}P$, CoP_v and $Co_{1-x}P_v$ in the non-Faraday region of 1.05 V-1.15 V vs. RHE with various scan rates (20, 40, 60, 80 and 100 mV s^{-1}) for OER in 0.5 M H_2SO_4.

References

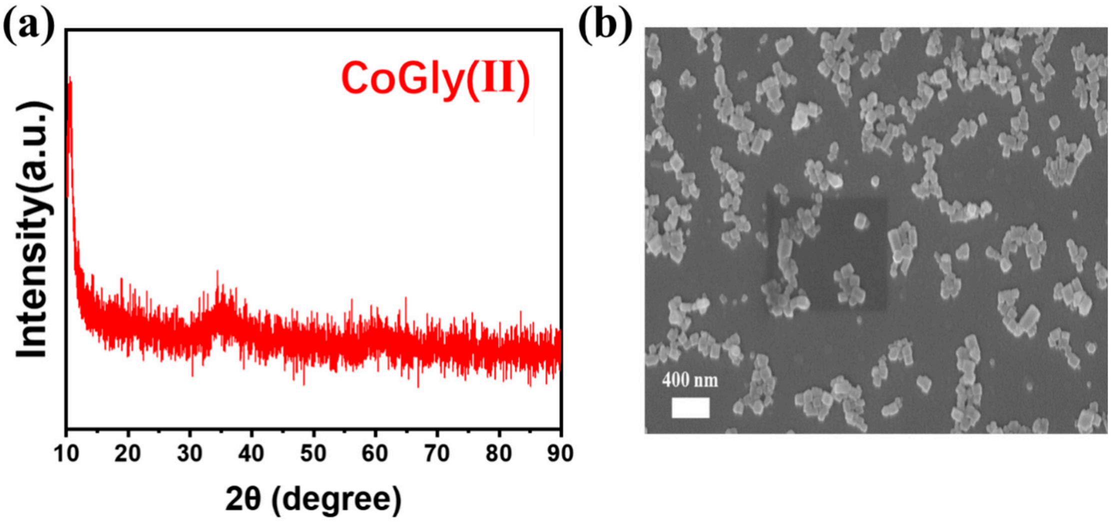

Figure S1. (a) XRD pattern and (b) SEM image of CoGly.

Table S1. EDX result of $Co_{1-x}P_{v}$.

Element	Wt%	Wt% Sigma
O	28.85	0.39
C	54.19	0.63
Co	10.02	0.25
P	6.94	0.20
Total:	100.00	

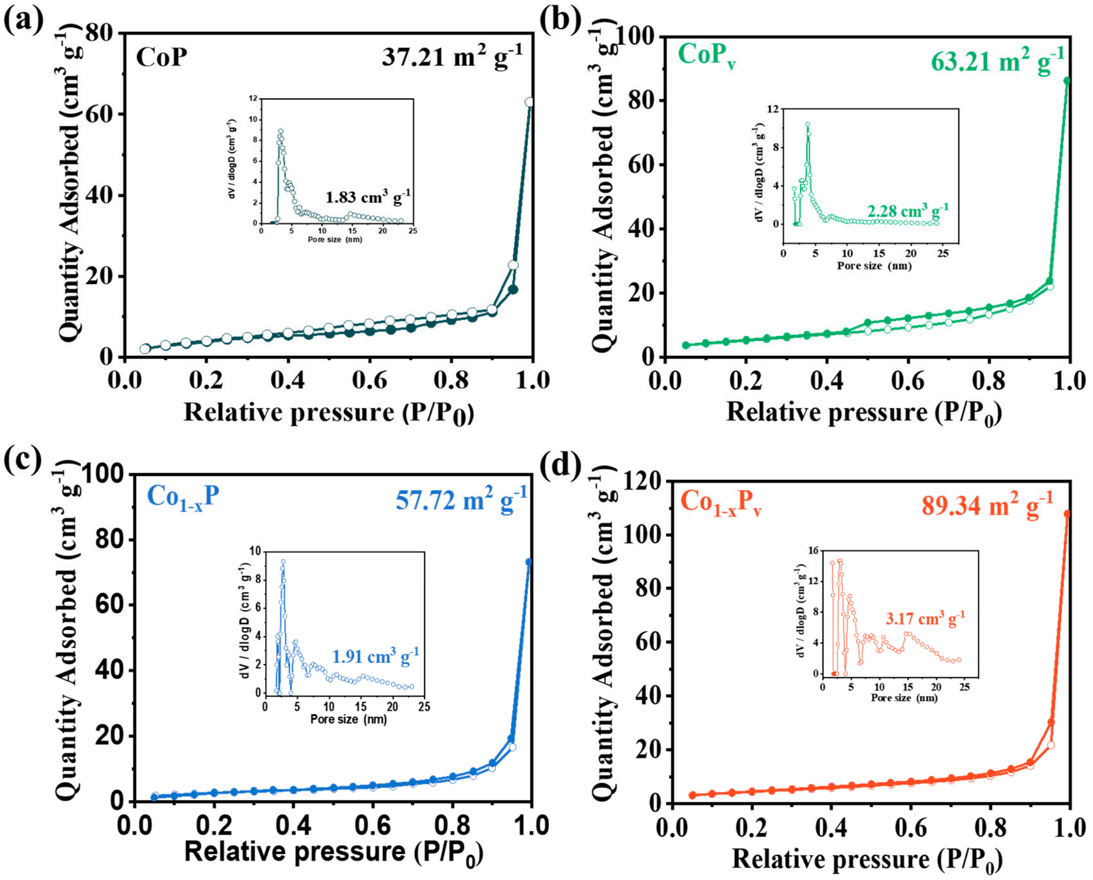

Figure S2. The Nitrogen adsorption-desorption isotherms of (a) CoP, (b) CoP_v, (c) $Co_{1-x}P$ and (d) $Co_{1-x}P_v$, where the inserts are the relevant pore size distribution.

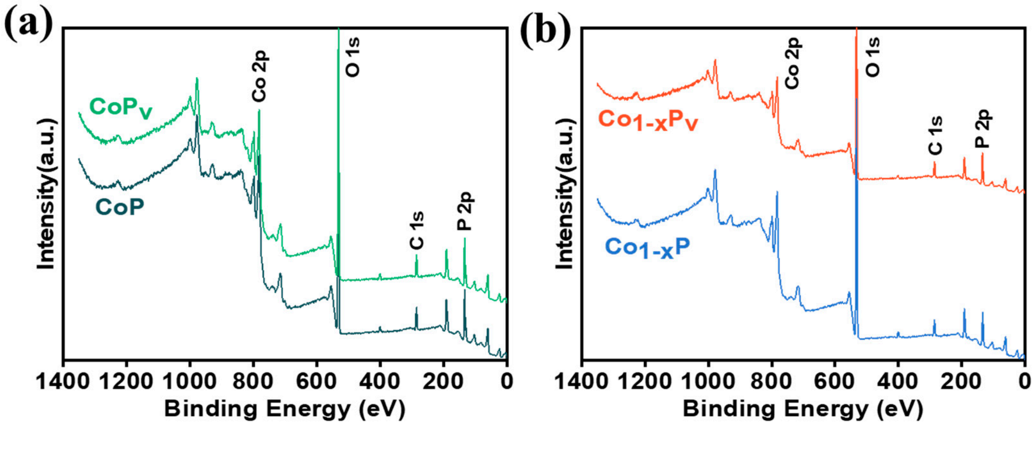

Figure S3. The wide-scan XPS spectrum of (a-b) CoP, CoP_v, $Co_{1-x}P$, $Co_{1-x}P_v$.

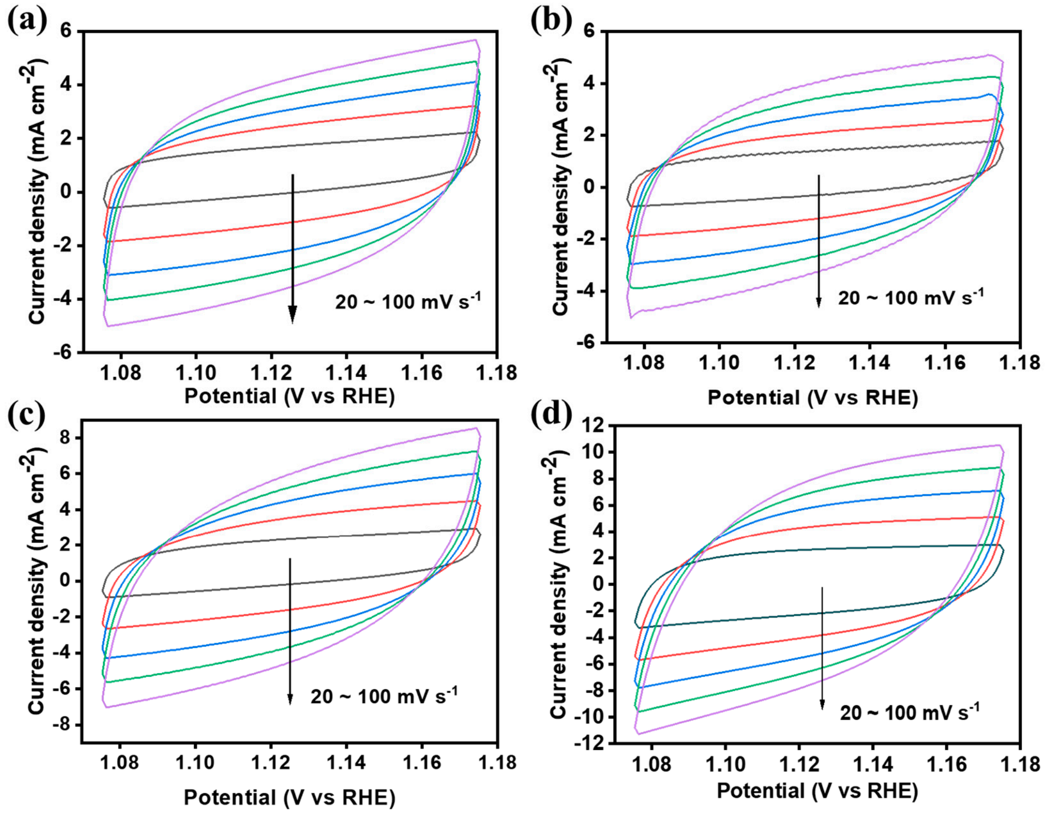

Figure S4 CV curves of (a-d) CoP , $Co_{1-x}P$, CoP_v , $Co_{1-x}P_v$ in the non-Faraday region of 1.075 V-1.175 V vs. RHE with various scan rates (20, 40, 60, 80 and 100 mV s^{-1}) for OER in 1.0 M KOH.

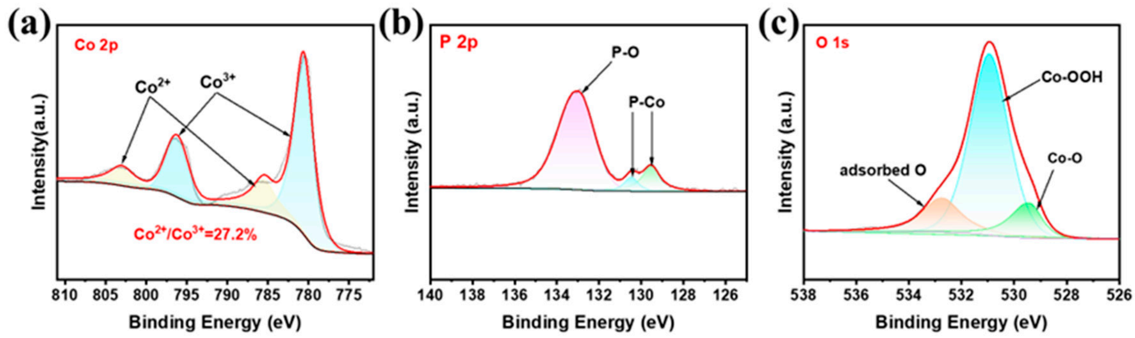

Figure S5 XPS spectrums of (a) Co 2p (b) P 2p and (c) O 1s of $Co_{1-x}P_v$ after i-t test[1].

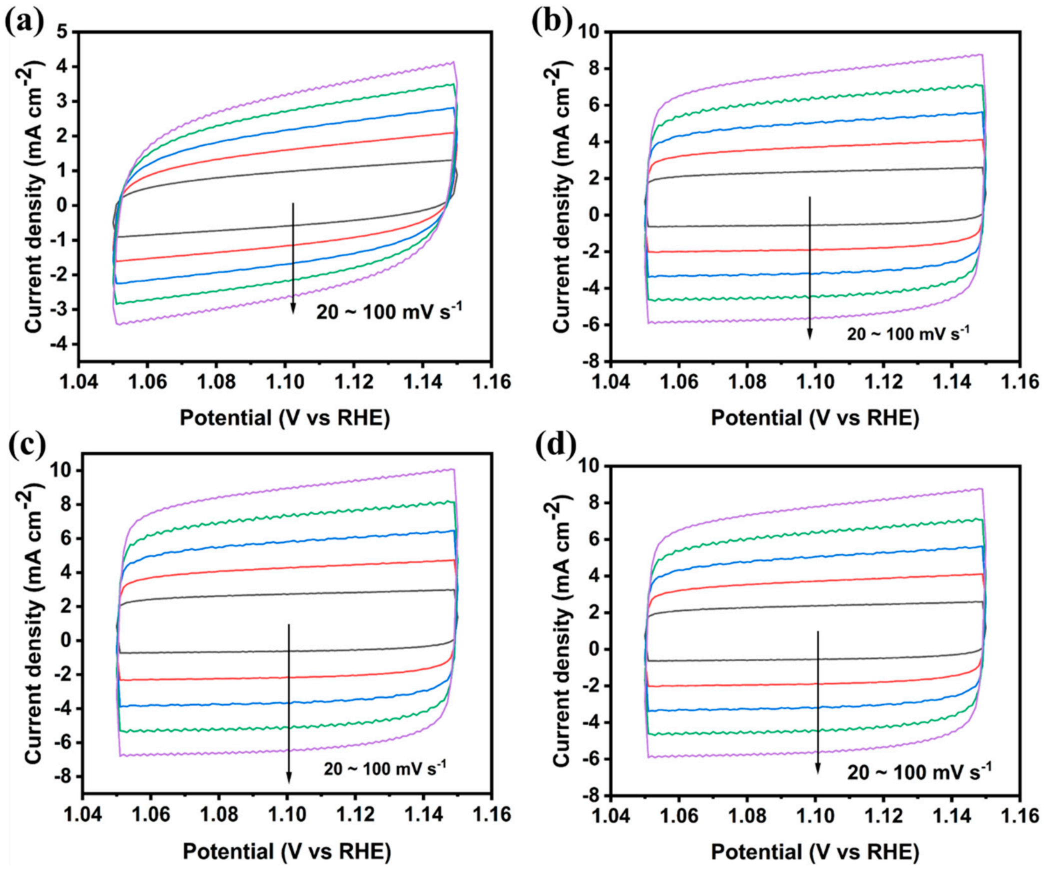

Figure S6 CV curves of (a-d) CoP , $Co_{1-x}P$, CoP_v and $Co_{1-x}P_v$ in the non-Faraday region of 1.05 V-1.15 V vs. RHE with various scan rates (20, 40, 60, 80 and 100 mV s^{-1}) for OER in 0.5 M H_2SO_4

References

1. Thiyagarajan, D.; Gao, M.Y.; Sun, L.; Dong, X.C.; Zheng, D.H.; Wahab, M.A.;Will, G.; Lin, J.J. Nanoarchitectured porous Cu-CoP nanoplates as electrocatalysts forefficient oxygen evolution reaction. *Chemical Engineering Journal* **2022**, *432*,doi:10.1016/j.cej.2021.134303.
